# Ecotoxicity of the Adipate Plasticizers: Influence of the Structure of the Alcohol Substituent

**DOI:** 10.3390/molecules26164833

**Published:** 2021-08-10

**Authors:** Irina Nikolaevna Vikhareva, Guliya Karamovna Aminova, Aliya Karamovna Mazitova

**Affiliations:** Applied and Natural Sciences Department, Ufa State Petroleum Technological University, 450080 Ufa, Russia; aminovagk@inbox.ru (G.K.A.); elenaasf@yandex.ru (A.K.M.)

**Keywords:** adipate plasticizer, biodegradable, environmental damage, esterification, polyvinyl chloride

## Abstract

A significant increase in the production of plastic materials and the expansion of their areas of application contributed to the accumulation of a large amount of waste of polymeric materials. Most of the polymer composition is made up of plasticizers. Phthalate plasticizers have been recognized as potentially hazardous to humans and the environment due to the long period of their biodegradation and the formation of persistent toxic metabolites. It is known that the industrial plasticizer dioctyl adipate is characterized by reduced toxicity and a short biodegradation period. The paper describes the synthesis of a number of new asymmetric esters based on adipic acid and ethoxylated butanol by azeotropic esterification. The receipt of the products was confirmed by IR spectra. The physicochemical properties of the synthesized compounds were investigated. The glass transition temperatures of PVC composites plasticized with alkyl butoxyethyl adipates were determined using DSC analysis. The ecological safety of esters was assessed by the phytotesting method. Samples of adipates were tested for fungal resistance, and the process of their biodegradation in soil was also studied. It is shown that the synthesized esters have good plasticizing properties and are environmentally safe. When utilized under natural conditions, they can serve as a potential source of carbon for soil microorganisms and do not form stable toxic metabolites; therefore, they are not able to accumulate in nature; when the plasticizers under study are disposed of in the soil, toxic substances do not enter.

## 1. Introduction

Industrial development is inextricably linked with the expansion of the range of chemicals used. The increase in the volume of chemicals applied is the reason for the steady growth of chemical hazards for the environment and humans.

A few decades ago, chemical production wastes were simply dumped into the environment. At the same time, it was believed that gaseous substances quickly dissipate in the atmosphere and liquids partially dissolve in water and are carried away from the places of release. Although solid products accumulate to a large extent in certain areas, the potential hazard of industrial emissions was considered to be low. In general, the use of chemicals gave an economic effect that many times exceeded the damage caused by toxicants to nature, which, in principle, was not taken into account.

However, already in 1962, Rachel Carson in the book “Silent spring” described the influence of anthropogenic factors on living systems, which is not always safe, and progress is associated with global risks: “No one yet knows what the ultimate consequences may be” [1,2]. Under public pressure, government legislation has emerged to regulate the release of xenobiotics. 

More than 150 million tons of synthetic polymers on a petrochemical basis, characterized by a long period of biodegradation, are produced annually all over the world [3]. Most of them are used for the production of disposable consumer products, such as food packaging. During the operation of plastic products and violation of the rules for their disposal, today about 8.3 million tons of plastic have entered the environment [4].

Environmental pollution by synthetic polymers is considered the most important environmental, economic and social threat. The widespread plastic is found in all environments and affects the biota of the planet. Since most polymeric materials do not decompose when recycled in the environment at the end of their life cycle and are quite resistant to environmental influences, pollution by waste polymeric materials is closely related to other global processes and has long-term consequences [5].

The main amount of plastic enters the soil in a variety of ways: With household waste, when using plastic mulch in agriculture, when irrigating with plastic-contaminated water, during degradation of the road surface, when transferring plastic fragments by air [6,7,8,9].

Relatively favorable conditions for the degradation of plastic materials are formed on the soil surface: direct ultraviolet radiation, oxygen availability and relatively high temperatures. In addition, agricultural tillage contributes to the destruction of large fragments to microplastics, and soil microorganisms accelerate the biodegradation of plastics [10,11,12,13]. Nevertheless, according to researchers’ estimates, the destruction of polymer waste in soil takes a long period of time.

The degradation of plastic entering the soil is determined by the ability of the soil environment for self-purification and is carried out mainly by living organisms of the soil biota [14,15]. In this case, soil animals perform the most important functions: Regulation of the activity of microorganisms, mechanical destruction of organic fragments, their movement in the soil profile, and the formation of this profile [16,17,18,19].

The degradability of polymeric materials in the environment and their danger to living organisms are determined by the chemical composition, size, shape of particles, the presence of perforation, the degree of porosity, the presence of a filler, etc. [20]. In the literature, a negative impact, lack of influence, and even stimulation of some processes and vital functions of soil-living organisms are noted [21]. Mostly investigated are the health damage or changes in populations due to the physical presence of plastic particles in the environment or food of soil animals. 

Modern polymeric materials are a complex mixture of polymers, residual monomers and various additives. Of the 50 main types of synthetic polymers, more than 60,000 polymer compositions have been created [22], and only about 20 of them have found wide application. The most used plastic polymers are polypropylene (PP; 16%), low density polyethylene and linear low density polyethylene (LDPE, LLDPE; 12%), polyvinylchloride (PVC; 11%), high density polyethylene (HDPE; 10%), and polyethylene terephthalate (PET; 5%), which in total account for more than 50% of total plastics usage [4]. Much less is known about the effect on animals of various additives included in the composition of polymeric materials, for example, plasticizers or flame retardants [23,24]. Many additives, for example, phthalates, alkylphenols and polybrominated diphenyl ethers, are dangerous for the biota and can cause disruptions in the synthesis and functioning of hormones [25].

In the composition of PVC materials, plasticizers account for up to 50%. These are widely used industrial chemicals that impart elasticity, processability and some specific properties to synthetic polymers. Plasticizers enter the environment during the production, manufacture, use and disposal of polymer products. For this reason, these additives are one of the main potential sources of environmental pollution [26,27,28,29].

Phthalates and adipates, the most commonly used plasticizers, are hydrophobic and lipophilic and can accumulate in the soil. In this regard, the need arises for an accurate toxicological assessment of these compounds. There is some analogy between the physiological and toxicological properties of substances similar in chemical nature. In addition, when studying the toxicity of plasticizers, it is necessary to take into account the toxicity of the substances forming them. The type of the forming acid, like the alcohol radical, has an effect on the physiological and toxicological properties of plasticizers. For example, it is known that, in the series of o-phthalic acid esters, an increase in toxicity manifests itself with a decrease in the number of carbon atoms in the alcohol radical [30]: diisooctyl phthalate < di (2-ethylhexyl) phthalate < dibutyl phthalate < diethyl phthalate < dimethyl phthalate.

Polyvinyl chloride containing ester plasticizers is highly susceptible to microbiological attack. Microscopic fungi and bacteria use similar plasticizers as a carbon source. However, it is known that some phthalates, such as diethylhexyl phthalate (DOP), negatively affect the reproductive organs by acting as a mimic of the sex hormone estrogen [31,32,33], which has contributed to the limited use of phthalate plasticizers. Non-toxic, biodegradable PVC adipate plasticizers are a safe alternative. Ecotoxicity studies of the industrial plasticizer dioctyl adipate have confirmed its safety for living organisms [34,35,36].

In this regard, we obtained asymmetric esters of adipic acid and ethoxylated butanol, investigated the possibility of their use as PVC plasticizers, and also studied their environmental safety.

## 2. Results

### 2.1. Synthesis of Butoxyethanol

**Scheme 1:**
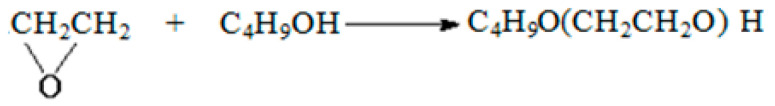
Synthesis of butoxyethanol.

Ethoxylation of alcohols has been well studied [37,38] (Scheme 1). The synthesis and characteristics of the product are described in our previous works [39,40].

Butoxyethanol with a degree of ethoxylation n = 1: density—0.9648, refractive index—1.4267, molecular weight (calculated)—118, yield—90.5%, reaction time—0.5 h.

### 2.2. The Synthesis of Esters of Adipic Acid and Butoxyethanol

**Scheme 2:**
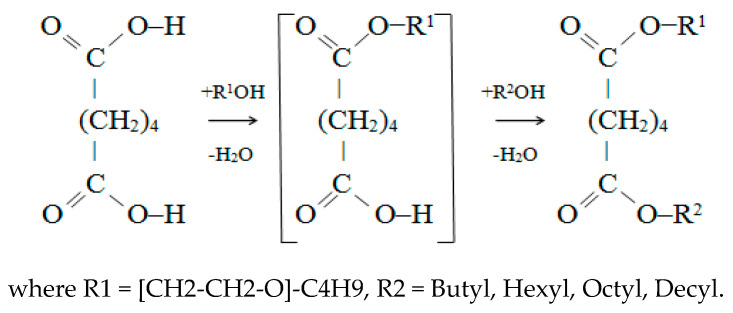
Synthesis of unsymmetrical esters of adipic acid.

The synthesis of unsymmetrical adipates of butoxyethanol was carried out by two-stage esterification of adipic acid in one reaction volume (Scheme 2). Initially, to obtain a monoester, a higher molecular weight alcohol was loaded into the reaction mass in an equimolar ratio of the starting reagents. To facilitate the removal of the released water, the synthesis was carried out in a toluene medium, the reaction mass was bubbled with an inert gas. The synthesis time was controlled by the acid number of the esterificate. Then diester was obtained. For this, another alcohol was loaded into the reactor in the ratio acid: alcohol = 1:1.2. Isolation of target esters was carried out according to the method described in [41]. Target esters were obtained with a yield of at least 80%.

The physicochemical properties of the obtained adipates are presented in the Table 1.

### 2.3. IR Spectra

The spectra of the synthesized esters lack an absorption band in the range of 1685–1687 cm^−1^, which is characteristic of stretching vibrations of the carbonyl group in associates of aliphatic carboxylic acids. In the synthesized esters, the stretching vibrations of the carbonyl group are shifted to the high-frequency region and appear as a strong characteristic band corresponding to the ester bands in the 1737 cm^−1^ region. There are also absorption bands that refer to the vibrations of the C-O-C ester fragment—1176 cm^−1^ for butyl butoxyethyl adipate, 1175 cm^−1^ for hexyl butoxyethyl adipate, 1173 cm^−1^ for octyl butoxyethyl adipate, 1173 cm^−1^ for decyl butoxyethyl adipate.

### 2.4. Determination of Glass Transition Temperatures

A decrease in the glass transition temperature with the introduction of a plasticizer is an important criterion for assessing the effectiveness of its plasticizing action.

The glass transition temperature was determined by DSC on a DSC-1 instrument (Mettler Toledo) at a heating rate of 2 K/min. The transition of a polymer from a glassy to a highly elastic state is accompanied by an increase in the heat capacity of the polymer, which is reflected in the form of a characteristic break (step) on the DSC curve. The Tg value was found in the middle of the step corresponding to this transition.

**Figure 1:**
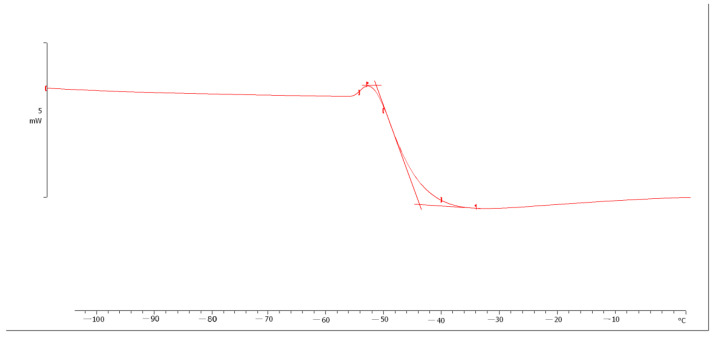
DSC thermogram of PVC composition with BBEA.

**Figure 2:**
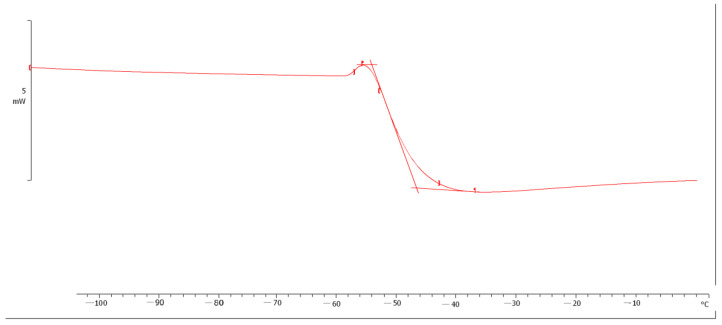
DSC thermogram of PVC composition with DBEA.

To assess the effect of plasticizers on the glass transition temperature, plasticized PVC compositions with a plasticizer content of 50 ppm were used for 100 ppm PVC (Figure 1 and Figure 2). The DSC thermograms of HBEA and OBEA look similar.

Table 2 shows the glass transition temperatures of PVC compositions with the content of the obtained alkyl butoxyethyl adipates.

### 2.5. Study of the Environmental Safety of Plasticizers by the Phytotesting Method

Within a month, we observed the growth of plants on a control soil sample and on soil samples, into which the obtained plasticizers were introduced in an amount of 1 g/dm^3^. For the selected biological objects, morphological parameters were determined and calculations were made for seed germination and germination energy for each phytotest system.

According to these indicators, the average soil toxicity and the effect of the studied additives on plant growth were evaluated when the samples were kept for one month. The phytotesting results are shown in the Table 3.

Radish and watercress were selected as indicator plants. The choice of these crops is due to the rapid germination of their seeds and high germination rate, which is noticeably reduced in the presence of pollutants; shoots and roots under the influence of toxic substances undergo morphological changes (growth retardation, curvature of shoots, reduction in root length, etc.).

In the control sample, the value of the average soil toxicity is 1.29, which corresponds to the stimulation of the growth and development of rasters. In experimental samples, the value of the test function fluctuates depending on the tested ester and gradually decreases with the elongation of the adipate molecule, that is, the observed toxicity of the soil sample with the ester used increases slightly. However, utilization of even the longest molecule from the entire range, decyl butoxyethyl adipate, does not have an adverse toxic effect on the growth and development of each phytotest system. Thus, the value for BBEA and HBEA corresponds to the norm: The factor does not significantly affect the development of test objects. The magnitude of the test functions is at the control level. The values for OBEA and DBEA correspond to low toxicity: There is a degree of decrease in test function compared to control.

### 2.6. Study of the Fungal Resistance of Alkyl Butoxyethyl Adipates

Table 4 shows the results of studies of the obtained alkyl butoxyethyl adipates for fungal resistance. 

Analysis of the results showed that all the obtained asymmetric esters of adipic acid and ethoxylated alcohol during the experiment time (28 days) showed the ability to serve as a food source for microscopic fungi: A significant overgrowth of fungal mycelium was observed in all samples. However, the mushroom resistance of OBEA and DBEA esters is slightly higher.

### 2.7. Biodegradation of Alkyl Butoxyethyl Adipates in Soil

**Figure 3:**
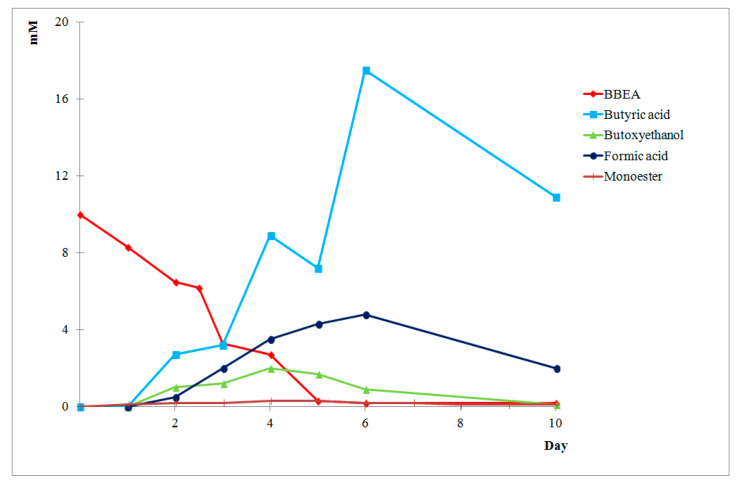
Biodegradation of BBEA in soil.

**Figure 4:**
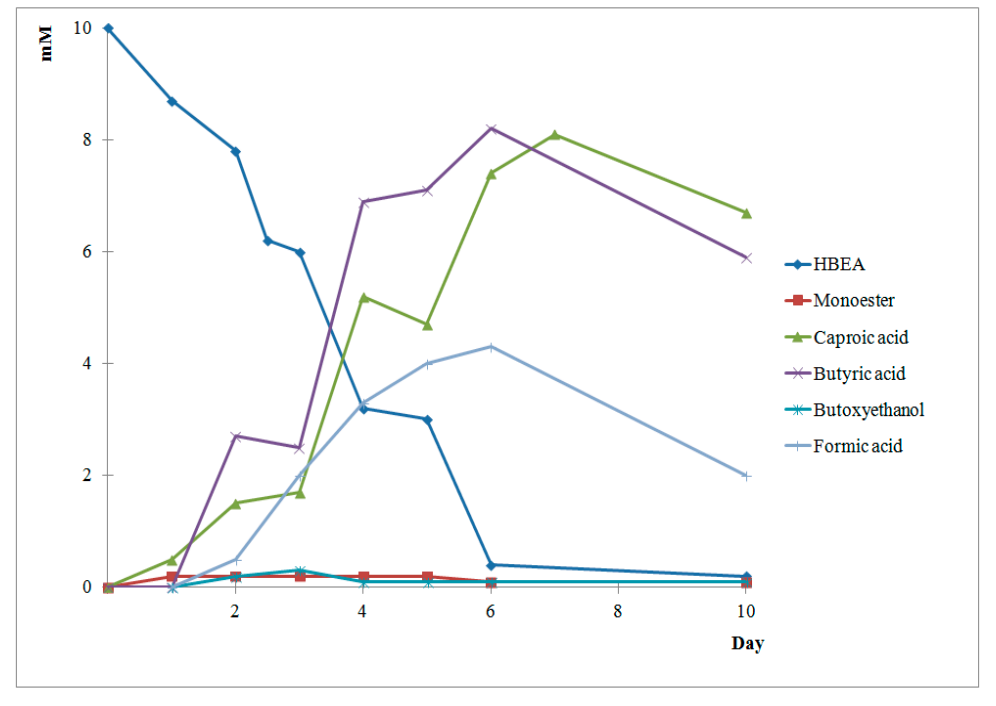
Biodegradation of HBEA in soil.

**Figure 5:**
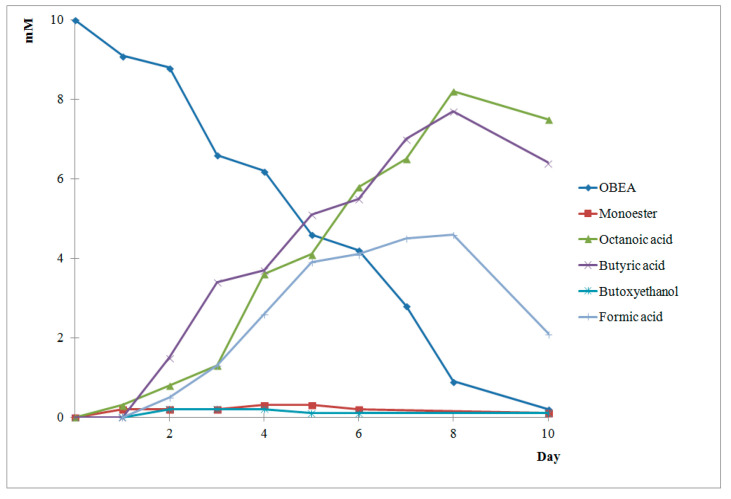
Biodegradation of OBEA in soil.

**Figure 6:**
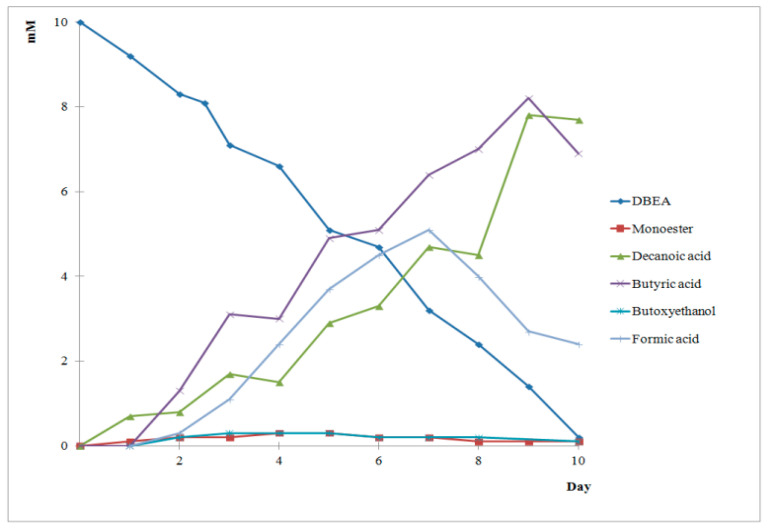
Biodegradation of DBEA in soil.

Biodegradation of the synthesized esters was tested in soil (Figure 3, Figure 4, Figure 5 and Figure 6).

The starting esters under conditions of natural degradation are rapidly hydrolyzed, and monoesters were found only in trace amounts. In the process of decomposition, a gradual accumulation of the corresponding alcohols is observed; however, butoxyethanol completely biodegrades within four days, and the corresponding aliphatic alcohols are oxidized within 5–10 days, depending on the length of the hydrocarbon chain. The figures do not show the alcohols formed during biodegradation, since these compounds are quickly subjected to further attack by microorganisms with the formation of the corresponding acids.

## 3. Discussions

Polymeric materials and products made from them are widespread. A large share in the composition of PVC composites is occupied by plasticizers, the characteristics of which determine the properties of the composites.

The most commonly used plasticizer, dioctyl phthalate, has a potentially hazardous environmental impact. A safe alternative to phthalates should have good plasticizing properties, be biodegradable upon disposal and safe during use, and also not contribute to the accumulation of toxic metabolites that contribute to the disruption of the biosphere balance.

Evaluation of the plasticizing properties of the obtained adipate plasticizers was carried out using the glass transition temperature. A decrease in the glass transition temperature due to the introduction of a plasticizer is one of the criteria for assessing the effectiveness of its plasticizing action. A decrease in the glass transition temperature of a polymer with the introduction of a plasticizer makes it possible to expand the temperature range of its highly elastic state. In addition, as a result, a decrease in the viscosity of polymer melts makes it possible to significantly facilitate their processing.

Studies have shown that all the obtained alkyl butoxyethyl adipates effectively reduce the glass transition temperature of PVC-based composites. Moreover, the lengthening of the chain of the alkyl substituent increases the temperature range of the highly elastic state of the obtained composites. Thus, the long group of the alcohol substituent more effectively prevents the interaction of the long chains of PVC, providing greater flexibility and ductility.

Since adipate plasticizers in PVC compositions are not bonded to the polymer by a chemical bond, these additives during operation and under the influence of certain factors tend to migrate from the film. In this regard, it is important that the developed plasticizers have reduced toxicity and do not form toxic metabolites in the process of biodegradation.

**Scheme 3:**
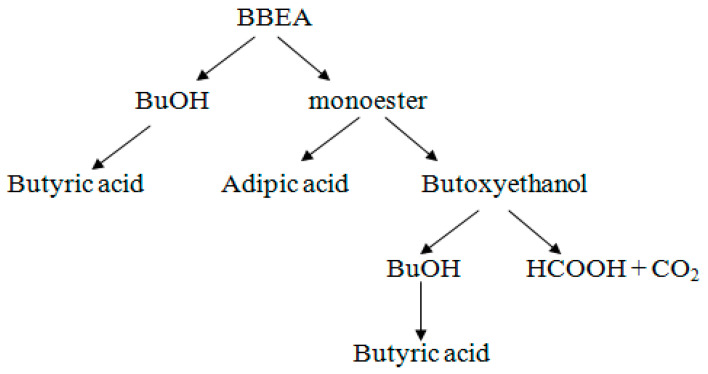
Path of butyl butoxyethyl adipate biodegradation.

Evaluation of the biodegradation of the obtained alkyl butoxyethyl adipates showed that the decomposition path of diester plasticizers includes sequential hydrolysis of ester bonds through the formation of a monoester of adipic acid. The presence of the latter was observed only in trace amounts. This is probably due to the same reactivity of both carboxyl groups of adipic acid. Determination of the stability of the monoester in such experiments is important, since it is believed that it is the monoester of the commercial plasticizer DEHP that exhibits toxicity and endocrine activity [42,43,44,45,46,47]. Scheme 3 shows the biodegradation pathway for butyl butoxyethyl adipate. Other adipates obtained undergo biodegradation in a similar manner to form the corresponding alcohols and acids.

Adipic acid, butoxyethanol, and the corresponding aliphatic alcohols—butanol, hexanol, octanol, and decanol—were found as further metabolites determined in the course of the study. With the lengthening of the chain of the alcohol being formed, the rate of hydrolysis during biodegradation decreased, and decanol was formed most slowly in the system. Thus, lengthening the chain of the ester molecule limits the rate of degradation. This is probably due to the difficulty of penetration of the ester molecule through the cell membrane of microorganisms that carry out the process, that is, shorter molecules are more actively processed by organisms present in the soil.

On the other hand, the long chain of the alcohol radical inhibits oxidation, which makes it a poor source of nutrition for microorganisms and leads to the accumulation of octanoic and decanoic acids.

Further, in the studied samples, the corresponding acids were determined as biodegradation products.

The resulting butoxyethanol also continues to undergo biodegradation to form butanol and formic acid as metabolites. 

The results obtained for the determination of the biodegradation products of the synthesized esters are in good agreement with the results of the study of the ecotoxicity of the products formed during the degradation of samples in the soil, which was carried out using the biotesting method.

Ecological and toxicological control using biotesting methods makes it possible to assess and predict the impact of anthropogenic load on biological systems [48,49]. Such an assessment is necessary to predict the prospects of biodegradable polymer composites. Biotesting methods based on the response of living organisms to the negative impact of pollutants are characterized by efficiency, speed of experiments, good reproducibility and reliability of results.

Phytotesting was carried out using watercress (Lepidium sativum) and radish (Raphanus sativus) as indicator plants. The choice of these crops is due to the rapid germination of their seeds and high germination rate, which is noticeably reduced in the presence of pollutants; shoots and roots under the influence of toxic substances undergo morphological changes (growth retardation, curvature of shoots, reduction in root length, etc.) [50]. 

Based on the toxicity rating scale and the results obtained during the disposal of the studied plasticizers in the soil, a different picture is observed when using various alcohols as a carbon source to support the growth of soil microorganisms.

When utilizing butyl butoxyethyl adipate in the soil, the stimulation of the growth of radish and watercress slightly differs from the control soil sample, the value of the test reaction in the experiment is slightly lower than the control value. With an increase in the length of the ester molecule, the magnitude of the test reaction in the experiment decreases, but the resulting metabolites do not adversely affect the stimulation of growth.

Consequently, when the studied plasticizers are utilized in the soil, toxic substances do not enter the soil and the growth of indicator plants is preserved, which confirms their ecotoxicological safety.

## 4. Materials and Methods

### 4.1. Materials

Adipic acid (purity 99.8%) was purchased from Radici Group, Selbitz-Hochfranken, Bavaria, Germany. Butanol (purity 99.7%), hexanol (purity 98%), octanol (purity 99%) and decanol (purity 99%) were purchased from The Company “Rearus”, (Moskow, Russia). Ethylene oxide is a solid with a purity of 98.2%, was purchased from ECOTECH Chemical Components Plant, Moscow, Russia. Sodium hydroxide was purchased from Joint Stock Company “Caustic,” Sterlitamak, Russia, it is a white solid with a purity of 98.2%. *P*-toluene sulfonic acid was purchased from Component-Reagent, Moscow, Russia, it is a white solid, with a purity of 95%. Toluene was purchased from Public Joint-Stock Company “Joint-Stock Oil Company Bashneft”, Ufa, Russia. It is a colorless liquid with a characteristic smell and a purity of 99.5%. Polyvinyl chloride (Joint Stock Company “Caustic,” Sterlitamak, Russia): We used industrial samples of suspension polyvinyl chloride PVC 7059M. Suspension polyvinyl chloride made by suspension polymerization, with a K value from 70 to 73, with a bulk density from 0.45 to 0.55 g/cm^3^, with a residue after sieving on a sieve with a mesh N 0.0063–95%, for the manufacture of plasticized products.

### 4.2. Synthesis Methods

#### 4.2.1. Synthesis of Ethoxylated Butanol

Ethoxylated butanol was obtained by the method described earlier [39]. Briefly, the calculated amount of alcohol and sodium hydroxide catalyst in the flask was heated to 110–120 °C, then purged with nitrogen to remove air, then prepared ethylene oxide was gradually introduced. The required temperature of the reaction mixture was maintained within the specified interval for 1 h and then cooled to room temperature. The catalyst was neutralized, the resulting mass was filtered and the light fraction was distilled off from the reaction mixture, boiling up to 50 °C at 10 mm Hg. 

To make sure that the compound was suitable for further work, its density, refractive index and molecular weight were determined. The purity of butoxyethanol was determined by HPLC.

Butoxyethanol is a colorless oily liquid soluble in water. The yield was 106.8 g (90.5% of theoretical).

#### 4.2.2. The Synthesis of Esters of Adipic Acid and Ethoxylated Butanol

All four unsymmetrical adipates were obtained according to the previously described method [40]. Briefly, 150 mL of toluene, 1 mol of ethoxylated alcohol, 3.0 g (1% by weight) of p-toluene sulfonic acid and 146 g (1 mol) of adipic acid were charged into the reaction flask and the temperature was slowly raised to 90–95 °C. Stirring was continued for 1.5 h. The end of the reaction was determined by the acid number of the esterificate.

Then was esterified with aliphatic alcohol (1.2 mol). Boiling was continued for 2–3 h. The reaction mixture was cooled and allocated target ester. All obtained adipates were the light clear liquids. The purity of esters was determined by HPLC.

The yield of butyl butoxyethyl adipate (BBEA) was 265.8 g (88.0% of theoretical).

The yield of hexyl butoxyethyl adipate (HBEA) was 290.7 g (88.1% of theoretical).

The yield of octyl butoxyethyl adipate (OBEA) was 318.3 g (88.9% of theoretical).

The yield of decyl butoxyethyl adipate (DBEA) was 341.2 g (88.4% of theoretical).

### 4.3. Methods of Analysis

#### 4.3.1. Analysis of Physicochemical Parameters of Plasticizer 

The analysis of physicochemical parameters of the compound was carried out in accordance with state standard 8728-88 Plasticizers. Specifications [41]. For this, the following indicators were determined: Acid number, ester number and mass fraction of volatile substances.

Determination of plasticizer density according to state standard 18329-2014 Liquid resins and plasticizers. Methods for determination of density [51].

#### 4.3.2. Characterization of Esters of Adipic Acid 

Samples of the products were analyzed by FTIR spectroscopy (KBr tablets), which were prepared according to a standard procedure. IR absorption spectra were recorded in the range 450–3700 cm^−1^ using an FTIR-8400S FTIR spectrometer (Shimadzu Corporation, Shimadzu, Japan) at room temperature. Resolution—4 cm^−1^, number of scans—20.

#### 4.3.3. Determination of Glass Transition Temperature

The glass transition temperatures of PVC composites containing the developed plasticizers were determined by differential scanning calorimetry using a DSC-1 instrument (Mettler Toledo, Greifensee, Switzerland).

The analysis was carried out in the temperature range from −100 to 100 °C in air. The measurements were carried out in a dynamic mode at a constant heating rate of 2 deg/min. The mass of the sample taken for measurements was 4–8 mg. For the analysis, aluminum crucibles with a volume of 40 μL were used. A weighed sample was placed in a crucible and sealed with a lid using a press. After quenching at −90 °C for 5 min, the sample was heated to 100 °C at a rate of 2 °C/min. The first heating cycle was used to remove any heat history. The glass transition temperature of the polymer was determined from the DSC curve obtained in the second heating cycle of the sample using the supplied software. Using the tangent method, the middle of the bend (step) on the curve was determined, which was taken as the glass transition temperature.

#### 4.3.4. Study of the Environmental Safety of Plasticizers by the Phytotesting Method

The ecotoxicity of the samples was assessed by the phytotesting method (Interstate standard 12038-84) [52]. For this, ester samples were mixed with soil in a ratio of 1 g of ester per 1 kg of soil and their effect on the germination and germination energy of seeds of test objects of radish (Raphanus sativus) and watercress (Lepidium sativum) was determined. The sprouting rate of emergence on the fifth day was calculated by the Formula (1):

B = a/b × 100 (%) (1)

where a—the number of germinated seeds;

b—the total number of seeds taken for the experiment.

The toxicity index of the evaluated factor (ITF) for each biological test object was calculated by the Formula (2):

ITF = TF_0_/TFk, (2)

where TF_0_—value of the registered test system in the experiment; 

TFk—value of the registered test system in control [53]. 

#### 4.3.5. Study of Fungal Resistance of Adipates

Tests for the ability of adipates to serve as a food source for micromycetes (fungal resistance) were carried out in accordance with Interstate standard 9.049–91. The essence of the method lies in the fact that the sample is infected with mold spores in water. Molds grow only on the nutrients contained in the material. samples of components are taken in 2–3 mL for liquid substances.

As test organisms, we used strains of 3 types of microscopic fungi, known as active destructors of various materials: *Aspergillus niger*, *Pénicillium* sp., *Paecilomyces* sp.

The fixtures and Petri dishes with the samples were transferred to the box and the surface of the samples was contaminated with a suspension of fungal spores by uniformly applying it with a spray bottle, preventing the droplets from merging. 

The contaminated samples were kept in a box at a temperature of 25 ± 10 °C and a relative humidity of up to 80% until the drops dried, but not more than 60 min. Samples and control Petri dishes were placed in a chamber, at the bottom of which water was poured. The camera was closed.

The tests were carried out at a temperature of 29 ± 2 °C and a relative humidity of more than 90%. No moisture condensation, forced ventilation or exposure to direct natural or artificial lighting were allowed in the chamber. The duration of tests in assessing the resistance of materials to the degree of development of fungi was 28 days with an intermediate inspection after 14 days. Every 7 days, the lid of the desiccator was slightly opened for 3 min to allow air to flow.

During intermediate examinations and at the end of the tests, the samples were removed from the chamber or desiccator, and examined with the naked eye in diffused light at an illumination of 2000–3000 lx and at a magnification of 56–60 times. Fungus resistance was assessed according to the intensity of development of fungi on samples according to a 6-point scale of the standard.

To determine the soil toxicity class when disposing of composite samples, the toxicity rating scale was used [54,55].

#### 4.3.6. Study of Biodegradation of Adipates in Soil

For testing, the obtained esters were mixed with soil in a ratio of 10 g of ester per 1 kg of soil. At certain intervals, samples of artificially contaminated soil were taken in an amount of 5 g and the content of metabolites in it was determined. For this, extraction was carried out with chloroform 3 times, 20 mL each. The solvent was evaporated. The analysis of the dry residue was determined by the HPLC method.

#### 4.3.7. High Performance Liquid Chromatography

The study of the composition of soil samples with utilized adipates was carried out by high performance liquid chromatography (LC-10 from SHIMADZU, Kyoto, Japan) in a reversed-phase mode. The separation of the mixture components was carried out on a column (150 × 4.6 mm) filled with a Separon-C18 sorbent with a particle size of 5 μm in an acetonitrile-water eluent system, taken in a 67/33 volumetric ratio. The eluent flow rate is 0.5 mL/min. A refractometric model detector (RIDK 101, Prague, Czech Republic) was used as a detector. The volume of injected samples was 10 μL. Quantitative analysis was performed using the absolute calibration method. The calibration solutions contained adipic acid, alcohols and esters.

### 4.4. Preparation of Film Samples

The following composition was prepared for testing: One-hundred parts by weight of PVC; 50 parts by weight of the plasticizer; 3 parts by weight of calcium stearate stabilizer.

To obtain test samples, all the ingredients of the PVC composition were mixed in a two-stage laboratory mixer for 60 min. To study the PVC composition, samples were obtained in the form of rigid and plasticized films. PVC film samples were obtained by rolling on laboratory rollers at temperatures of 165–175 °C for 5 min.

## 5. Conclusions

The analysis of the conducted studies showed that the obtained alkyl butoxyethyl adipates are environmentally friendly plasticizers. All esters showed good plasticizing properties, with an increase in the total molecular length within the investigated range improving the effective effect.

From the point of view of biodegradation, the elongation of the molecule of the developed compounds is a factor contributing to a decrease in the rate of hydrolysis of the ester bond and the subsequent oxidation of alcohol.

In terms of ecotoxicological assessment, the intermediate monoesters were found to be unstable compounds and were found in trace amounts.

Thus, the developed unsymmetrical esters of adipic acid and ethoxylated butanol can serve as a potential source of carbon for soil microorganisms, without being toxicants and without forming persistent toxic metabolites in the process of biodegradation.

## Figures and Tables

**Table 1:** Physicochemical properties of alkyl butoxyethyl adipates.

Ester	Indicators			
	Molecular Weight	Acid Number mg KOH/g	Ester Number mg KOH/g	d^20^_4_
BBEA	302	0.1	369	1.0452
HBEA	330	0.1	337	1.0636
OBEA	358	0.1	311	1.0819
DBEA	386	0.1	288	1.1003

**Table 2:** Glass transition temperatures of PVC compositions containing alkyl butoxyethyl adipates.

PVC Composition	Glass Transition Temperature, °C	∆T, °C
PVC	87.5	–
DOP	−37.0	124.5
BBEA	−48.7	136.2
HBEA	−49.2	136.7
OBEA	−49.7	137.2
DBEA	−50.2	137.7

**Table 3:** Results of determining the phytotoxicity of alkyl butoxyethyl adipates.

Sample		Indicators					
		Average Root Length, cm	Average Length of the Ground Part, cm	Seed Germination, %	Germination Energy for 5 Days, %	ITF	Average Soil Toxicity
Control	watercress	1.96	0.51	93	81	1.22	1.29
	radish	14.36	8.32	91	77	1.35	
BBEA	watercress	2.01	0.56	90	76	1.02	1.01
	radish	14.42	8.64	91	77	1.00	
HBEA	watercress	1.95	0.50	87	72	0.99	1.00
	radish	14.30	8.90	90	73	1.00	
OBEA	watercress	1.80	0.38	73	69	0.92	0.95
	radish	13.90	8.01	75	64	0.97	
DBEA	watercress	1.62	0.24	65	60	0.83	0.84
	radish	12.21	7.19	62	61	0.85	

**Table 4:** Fungal resistance of alkyl butoxyethyl adipates.

Ester	The Degree of Fouling in Points		
	*Aspergillus niger*	*Pénicillium funiculosum*	*Trichoderma lignorum*
BBEA	4	4	4
HBEA	4	4	4
OBEA	3	3	3
DBEA	3	3	3

## Data Availability

The data presented in this study are available on request from the corresponding authors.

## Sample Availability

Samples of the compounds butyl butoxyethyl adipate, hexyl butoxyethyl adipate, octyl butoxyethyl adipate and decyl butoxyethyl adipate are available from the authors.
